# Caprate Modulates Intestinal Barrier Function in Porcine Peyer’s Patch Follicle-Associated Epithelium

**DOI:** 10.3390/ijms20061418

**Published:** 2019-03-20

**Authors:** Judith Radloff, Valeria Cornelius, Alexander G. Markov, Salah Amasheh

**Affiliations:** 1 Institute of Veterinary Physiology, Department of Veterinary Medicine, Freie Universität Berlin, 14163 Berlin, Germany; judith.radloff@vetmeduni.ac.at (J.R.); valeria.cornelius@fu-berlin.de (V.C.); 2 Department for Biomedical Sciences, University of Veterinary Medicine, 1210 Vienna, Austria; 3 Department of General Physiology, St. Petersburg State University, Universitetskaya nab. 7/9, 199034 St. Petersburg, Russia; markov_51@mail.ru

**Keywords:** tissue barrier, tight junction, claudins, tricellulin

## Abstract

Background: Many food components influence intestinal epithelial barrier properties and might therefore also affect susceptibility to the development of food allergies. Such allergies are triggered by increased antibody production initiated in Peyer’s patches (PP). Usually, the presentation of antigens in the lumen of the gut to the immune cells of the PP is strongly regulated by the follicle-associated epithelium (FAE) that covers the PP. As the food component caprate has been shown to impede barrier properties in villous epithelium, we hypothesized that caprate also affects the barrier function of the PP FAE, thereby possibly contributing a risk factor for the development of food allergies. Methods: In this study, we have focused on the effects of caprate on the barrier function of PP, employing in vitro and ex vivo experimental setups to investigate functional and molecular barrier properties. Incubation with caprate induced an increase of transepithelial resistance, and a marked increase of permeability for the paracellular marker fluorescein in porcine PP to 180% of control values. These effects are in accordance with changes in the expression levels of the barrier-forming tight junction proteins tricellulin and claudin-5. Conclusions: This barrier-affecting mechanism could be involved in the initial steps of a food allergy, since it might trigger unregulated contact of the gut lumen with antigens.

## 1. Introduction

The gastrointestinal mucosa is one of the largest surface areas of the body and constantly interacts with possibly harmful substances or non-hazardous food components. The intestinal epithelial cells and the associated immune cells of the gut-associated-lymphocytic tissue (GALT) need to be able to distinguish the category of a molecule.

Peyer’s patches (PP) represent the dominating part of the GALT. They are structured into a germinal center that consists of macrophages, lymphocytes, and dendritic cells (DC) and a covering epithelial cell layer, namely the follicle-associated epithelium (FAE) [1]. The FAE of rat and pig has recently been characterized in detail, giving a greater insight into the barrier properties directly above the PP [2,3]. Generally, the FAE has a more porous basal membrane and contains fewer goblet cells, resulting in a thinner mucus layer, than the villous epithelium (VE) [4,5]. The FAE also includes microfold cells (M-cells), which are specialized for antigen translocation to the underlying cells of the PP [4]. The presentation of antigens can also be initiated via DC that reach through the epithelial cell layer into the gut lumen and directly sample bacteria. During this process, the barrier integrity is preserved, as DC also express tight junction (TJ) proteins [6]. Overall, the barrier of the FAE can be classified as tight when compared with the surrounding leaky VE, as sealing claudins such as claudin-1, -3, -4, -5, and -8 are predominantly expressed in PP samples of various species [2,3,7].

Numerous food components have been shown to influence barrier integrity in the VE, including the medium chain fatty acids laurate and caprate [8,9,10], the amino acids glutamine and glycine [11,12], and plant components quercetin and berberine [13,14], all which lead to a change of transepithelial resistance (TER) and paracellular permeability. In turn, this can enhance or limit the presentation of common food components to the immune cells of the PP, triggering or impeding the excessive formation of immunoglobulin E (IgE) [15]. The consequence is commonly known as food allergy.

Food allergies are defined as an adverse health effect arising from a specific immune response that occurs reproducibly on exposure to a given food [16]. With food allergies being more prevalent than ever, the need for an effective treatment or prevention mechanism is obvious [17]. This remains difficult for as long as the precise causes remain unidentified.

One of these causes might involve the barrier-weakening effect of the medium chain fatty acid caprate. Caprate has been shown to decrease the TER reversibly, leading to an increase of paracellular permeability for fluorescein and various fluorescein isothyocyanate (FITC)-dextrans (FD4, FD10) in the intestinal cell line HT29-B6 [8]. This can be attributed to the reversible decrease of the sealing TJ proteins claudin-5 and tricellulin, an effect opening the paracellular pathway [8]. Because of its reversible barrier-weakening properties, caprate is used as an absorption enhancer, improving membrane permeation for pharmaceutically active agents [18]. Since caprate is also contained in common foods, such as milk and coconut oil, it is widely consumed by most people, albeit in low concentrations [19].

Despite the numerous studies on the effect of caprate on intestinal barrier function [8,20,21], the effect of caprate on the FAE has not yet been investigated. Therefore, we have focused on the barrier-influencing properties of caprate on the FAE of the porcine PP and on the consequences with regard to gastrointestinal immunology.

## 2. Results

### 2.1. Cell Culture Study

Initial studies on the effect of caprate on the porcine intestinal barrier function were carried out by employing the Intestinal Porcine Epithelial Cell line-J2 (IPEC-J2) and 5 mM caprate.

**Figure 1:**
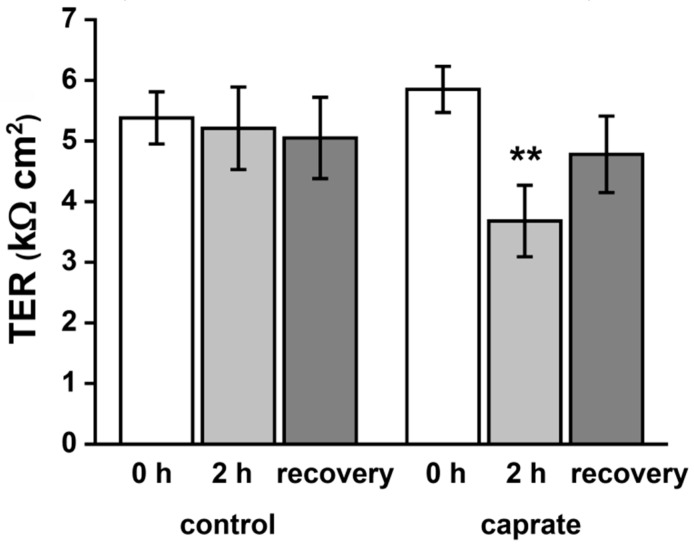
Transepithelial electrical resistance (TER) of IPEC-J2 cell monolayers. Incubation with 5 mM caprate leads to a reversible decrease of TER (** *p* < 0.01, *n* = 20).

Incubation of IPEC-J2 cells with 5 mM caprate led to a rapid and consistent decrease of barrier function (from 5.85 ± 0.38 kΩ to 3.68 ± 0.59 kΩ, ** *p* < 0.01, *n* = 20) when compared with the control group (not significant (n. s.), *n* = 20; Figure 1). This decrease was evident during the whole timespan of the experiment (120 min). After a recovery period of 24–48 h, TER values of the caprate group were comparable with control levels (4.78 ± 0.63 kΩ to 5.05 ± 0.67 kΩ, *n* = 20).

### 2.2. Ussing Chamber Experiments

The effect of caprate on PP tissue taken from adult pigs was analyzed by the Ussing chamber technique. Tissue was incubated with 5 mM caprate while TER values and paracellular permeability measurements for sodium fluorescein were carried out.

**Figure 2:**
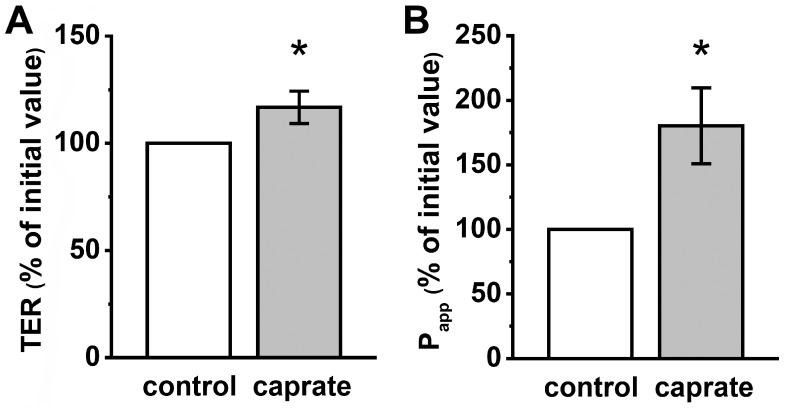
(**A**) Transepithelial electrical resistance (TER) and (**B**) apparent permeability (P_app_) of the paracellular flux marker fluorescein in porcine Peyer’s patches (PP). 5 mM caprate lead to markedly higher TER values (* *p* < 0.05, *n* = 11–12), and increased permeability for fluorescein (*n* = 8, * *p* < 0.05).

In contrast to the IPEC-J2 experiments, the TER of PP increased significantly after incubation of the tissue with caprate (240 min; 92.97 ± 4.94% to 116.73 ± 7.55%, * *p* < 0.05, *n* = 11–12) (Figure 2A). However, the permeability measurements of sodium fluorescein revealed a marked increase to 180.24 ± 29.52% (* *p* < 0.05, *n* = 8; Figure 2B).

### 2.3. Immunoblotting

**Figure 3:**
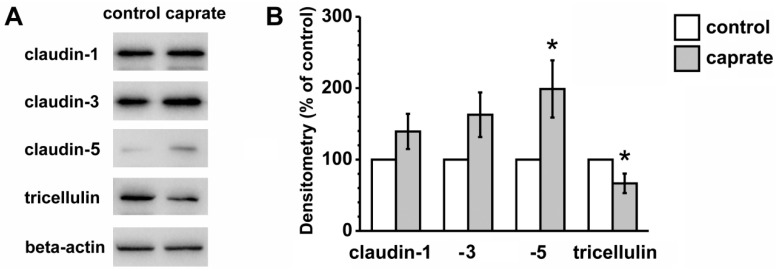
(**A**) Immunoblots and (**B**) densitometry of TJ proteins in PP tissue (control versus caprate). Densitometric analysis of TJ protein signals after caprate incubation reveals the lower expression of tricellulin, whereas stronger expression of claudin-5 can be detected (*n* = 6–8, * *p* < 0.05).

To account for the detected functional differences in TER, the expression levels of sealing TJ proteins were analyzed; the immunoblots revealed major changes (Figure 3). Whereas claudin-3 only showed a positive trend in expression levels (162.7 ± 31.23%, *p* = 0.064, *n* = 8), claudin-5 was significantly increased (198.82 ± 40.11%, *p* < 0.05, *n* = 8). Tricellulin on the other hand was significantly reduced (66.64 ± 13.69%, *p* < 0.05, *n* = 6). Sealing claudin-1 in PP tissue (139.32 ± 24.62%, *n* = 8) was not affected by caprate incubation.

### 2.4. Immunohistochemistry

**Figure 4:**
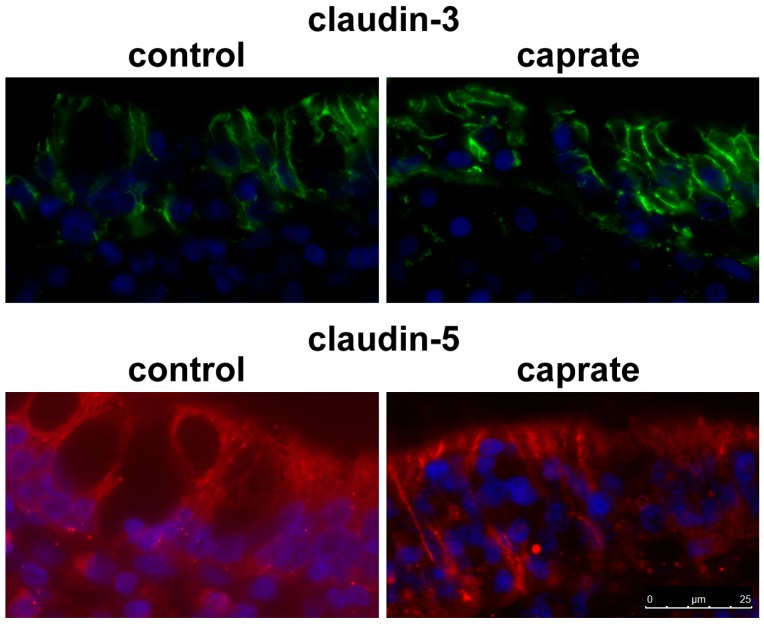
Immunohistochemistry. TJ proteins claudin-3 and claudin-5 are detectable as paracellular signals in the surface epithelium in PP tissue (bar: 25 µm).

To analyze the location of single TJ proteins, immunoflourescent staining of tissue specimens was performed following the Ussing chamber experiments. Immunohistochemistry of these self-same tissues revealed specific signals for TJ proteins in all tested PP tissue specimens, as shown for claudin-3 and claudin-5 (Figure 4).

## 3. Discussion

### 3.1. Suitability of a Porcine Model for Human Gastrointestinal Pathophysiology

Porcine models exhibit certain benefits when exploring molecular mechanisms of intestinal pathophysiological conditions. In 2007, Humphray et al. showed that 60% of the porcine and human genome sequence match [22]. The morphology and anatomy of the human gastrointestinal tract is comparable with that of a pig [23]. Moreover, the physiology of digestive processes and the subsequent absorption of nutrients are also similar [24,25]. Last but not least, the pig is the only commonly used laboratory animal that has comparable nutritional needs with humans, on account of it being classified as an omnivore [26].

Since its origin in 1989, the IPEC-J2 cell line has been extensively used for studies on the gastrointestinal barrier and the effects of nutrients. It represents one of the best cell lines of non-human origin for intestinal research because of its non-transformed properties [27,28]. Taking all of these aspects into account, we chose porcine intestinal epithelial tissue specimens and the IPEC-J2 cell line as promising models for studying the effects of caprate on barrier function with regard to immunologically relevant mechanisms, which might also provide a perspective as a model for porcine PP FAE.

### 3.2. Caprate in Modern Foods

Coconut oil is one of the main contributors of caprate in Western nutrition. Since it is currently regarded as a superfood, the intake of coconut oil, and therefore caprate, has increased tremendously. The health benefits of coconut oil include weight loss, cardioprotective effects, and improved immunity and memory [29]. The milk of various mammalian species is the other main source of caprate in current nutrition [19,30]. Existing data on the health benefits of milk include preventive effects against diabetes mellitus, cancer, hypertension, and obesity [31].

Even without the specific ingestion of caprate as an absorption enhancer, the daily intake of caprate can be high because of the recommended consumption of 2.5–3 servings of dairy products [32]. Digestion effects can be expectedly rather limited, as the medium chain fatty acid absorbed is non-modified, even devoid pancreatic enzymes [33]. Moreover, medium-chain triglycerides are degraded into fatty acids and the glycerol backbone, a mechanism adding to luminal caprate concentration. After absorption, the medium chain fatty acids are transferred into the portal vein for transport to the liver for hepatic metabolism [34]. However, prior to absorption, luminal caprate effects might even add up with effects of other bioactive compounds, e.g., laurate, which then might reach barrier-affecting concentrations earlier [9].

### 3.3. Effects of Caprate

Since foods containing caprate are consumed regularly, the effects of the fatty acid need to be brought into focus. Currently, caprate is used as an absorption enhancer to improve the bioavailability of pharmaceutically active agents [18]. A human in vitro cell culture experiment, employing the colonic HT-29/B6 cell line, has revealed the cellular mechanisms involving the selective removal of claudin-5 and tricellulin from the TJ [8]. Recent in vitro experiments have provided evidence of the antibacterial effect of caprate in porcine epithelial cells when challenged with *Salmonella enteritidis* or an enteropathogenic *Escherichia coli* strain [35]. Furthermore, caprate has also been shown to reduce body fat and therefore, body weight [36].

Data concerning the intestinal barrier function obtained in this study has shown that the effect of caprate can also occur in other species. Significantly lower TER values were recorded for the porcine IPEC-J2 cell line after caprate incubation (5 mM). This effect was reversible in both species (compare with [8] for human cells). However, transferring this functional result to the ex vivo setup using the Ussing chamber technique was not possible, as the TER markedly increased in PP tissue. This apparent discrepancy might reflect a limited suitability of the plain IPEC-J2 model for modeling the PP FAE. However, it might still provide a suitable matrix for, e.g., co-culturing experiments approaching a functional FAE model, in vitro.

Permeability measurements in PP tissue revealed a higher permeability for fluorescein, which can be compared with glucose in size (330 Da). A higher permeability for fluorescein, but also for FITC-dextran (4k, 10k), was observed after incubation with 10 mM caprate in HT-29/B6 cells [8].

Increased paracellular permeability might give rise to unregulated contact between the immune cells of the PP and food components. The most common foods that provoke allergic reactions are nuts, eggs, milk, soy, wheat, and shell fish [37]. The uptake of antigens within the PP is strictly regulated, as it is usually undertaken by specific binding to receptors, through specialized epithelial cells, the M-cells, or via the extending dendrites of DC belonging to the underlying PP [4,6,38]. An impairment of the intestinal barrier function would interrupt these controlled processes.

### 3.4. Caprate Effects on Tight Junction Protein Expression

The study has shown that claudin-5 and tricellulin have been removed from bi- and tricellular TJ through caprate incubation [8]. TJ are apicolateral epithelial cell membrane contacts determining paracellular permeability [39,40,41,42]. Claudin-5 is classically regarded as a sealing claudin, since it increases barrier properties by effects on TER [43,44].

Tricellulin was the first barrier-forming TJ protein to be found in tricellular TJ, and supression of tricellulin leads to the disruption of bicellular and tricellular TJ [45]. Overexpression of tricellulin increases barrier function and decreases the permeability of ions and larger solutes [46]. As caprate induces a reversible removal of claudin-5 and tricellulin from the TJ, functional results under caprate incubation can easily be attributed to structural changes of TJ formation.

The immunoblots after Ussing chamber experiments in our study also revealed changes in claudin-5 and tricellulin. Quantitative analysis of PP tissue samples showed an increased expression level of claudin-5 and decreased levels of tricellulin. These results suggest the differential sensitivity of the FAE towards the effects of caprate when compared with the intestinal epithelial monolayers that were used in the previously performed initial incubation experiment [8]. This is understandable when we keep the main task of the PP in mind. A structural change in TJ formation prevents the accidental passage of antigen to the underlying PP. Our immunohistological assessment has shown the paracellular localization of claudin-3 and claudin-5. Although a decrease of claudin-5 signals could not be observed in the current study, the overall increase of its expression level might have contributed to a compensation, in accordance with the observed increase of TER. This apparent inconsistency might also be explained by the different contribution of single tight junction proteins to transepithelial resistance and permeability of flux markers, as tricellulin is affecting the paracellular passage of molecules [46], whereas claudin-5 primarily affects the passage of ions [44]. However, our results indicate that it is possible to functionally uncouple resistance and solute permeability by affecting different tight junction proteins in parallel, which includes the possibility that an increased expression of a sealing protein such as claudin-5 does not necessarily provide an expected primary functional effect.

However, further studies, particularly in humans, are required to demonstrate that caprate may affect the susceptibility to the development of food allergies.

## 4. Materials and Methods

### 4.1. Cell Culturing and Experiments

The porcine jejunal cell line IPEC-J2 is derived from the jejunum of unsuckled piglets and is commonly regarded as an in vitro model for the analysis of the intestinal barrier function in both pigs and humans [27]. For routine cultivation, Dulbecco’s Modified Eagle Medium (MEM)/ Nutrient F 12 Ham’s (Biochrom, Berlin, Germany) was used as the medium basis, with 10% fetal calf serum, 1% penicillin-streptomycin, 5 ng /mL epithelial growth factor (Biochrom, Berlin, Germany) and insulin, transferrin and selenium (Life Technologies, Carlsbad, CA, USA) as supplements. Cells were passaged twice a week and fed every other day.

For experiments, cells (10^5^) were seeded on cell culture inserts with a pore size of 0.45 µm (Millicell, Darmstadt, Germany). The seeded inserts were placed into multiwell plates and cultivated for approximately 14 days until a functional barrier had formed. This was assessed via measurements of TER employing a chopstick electrode and an Epithelial Volt/Ohm Meter (EVOM) measuring device (World Precision Instruments, Hertfordshire, United Kingdom). Only filters that showed TER values above 2 kΩ were chosen for experimentation. Passages ranging from 9 to 15 were used in this experimental setup. TER values were measured initially in plain culture medium. Subsequently, the culture media was replaced with medium A (plain culture medium) or medium B (culture medium with 5 mM caprate). During the incubation period of 120 min, TER values were measured every 20 min, after which the medium was replaced with plain culture medium. Following a recovery period of 24–48 h, TER was measured again. The cells were kept at 37 °C during the recovery period.

### 4.2. Tissue Samples

Samples of the distal small intestine of 12 adult pigs were taken immediately after slaughter at a commercial slaughterhouse (Salzbrunn, Germany). PP tissue was transported in cold (4–6 °C) buffer solution containing (in mmol·L-1): Na^+^ (149.9), Cl^−^ (128.8), K^+^ (5), Ca^2+^ (1.2), Mg^2+^ (1.2), HCO_3_^−^ (25), H_2_PO_4_^2−^ (0.6), HPO_4_^−^ (1.2), and D-glucose (10). The pH was set to 7.4 before transport.

### 4.3. Ussing Chamber Experiments

Tissue samples were mounted in a common Ussing chamber setup (Mussler Scientific Instruments, Aachen, Germany), and data recording commenced after 45 min of preincubation with fluorescein, but only once electrophysiological values were stable. The incubation experiment itself was carried out under voltage clamp conditions. The experimental buffer contained (in mmol·L-1): Na^+^ (149.9), Cl^−^ (128.8), K^+^ (5), Ca^2+^ (1.2), Mg^2+^ (1.2), HCO_3_^−^ (25), H_2_PO_4_^2−^ (0.6), HPO_4_^−^ (1.2), and D-glucose (10). It was constantly gassed with 95% O_2_ and 5% CO_2_, resulting in a pH of 7.4. After 30 min, 5 mM caprate was added to the apical side. TER was recorded continuously throughout the overall incubation time of 4 h. Electrophysiological data are expressed in relation to the initial value before the addition of caprate.

### 4.4. Permeability Measurements

Once the tissue had been mounted in the Ussing chamber, fluorescein (100 µmol·L^−1^) was added to the apical side of each chamber. During the incubation experiment, permeability samples were taken every 60 min from the basolateral side. The volume that was taken was replaced with fresh buffer solution. Samples were measured photometrically at 514 nm by employing the EnSpire Multimode Plate Reader (Perkin Elmer, Waltham, MA, USA). Paracellular flux and permeability were then calculated by using the following equations.

c = (c_t−1_ × V_S_ + c_t_ × V_K_)/V_K_ (1)

J = (c_t_ − c_t−1_) × V_K_/∆t × A (2)

P_app_ = J/c_a_. (3)

In Equation (1), the measured concentration of fluorescein in the basolateral sample has to be corrected for the dilution that takes effect because of the replacement of the experimental buffer, with ct being the measured concentration, c_t−1_ the measured concentration of the previous period, V_S_ the sample volume, and V_K_ the volume of the Ussing chamber. Equation (2) gives the paracellular flux (J) for fluorescein, with ∆t being the duration of the measurement period, and A being the area within the Ussing chamber. Finally, the paracellular permeability can be calculated by using Equation (3), with c_a_ being the fluorescein concentration in the apical side of the Ussing chamber. Results were expressed as a fraction of the control values.

### 4.5. Protein Extraction and Quantification

Samples for protein extraction were taken out of the Ussing chamber and directly placed into liquid nitrogen. These samples were homogenized in a Tris-buffer containing in mmol·L -1: Tris (10), NaCl (150), Triton X-100 (0.5), SDS (0.1), and enzymatic protease inhibitors (Complete, Boehringer, Mannheim, Germany). After homogenization, samples were centrifuged for 1 min at 13,000 rpm (Eppendorf centrifuge 5418, Eppendorf AG, Hamburg, Germany). The supernatant was then cooled on ice for 30 min and retrieved after a second centrifugation step for 15 min at 15,000 g at 4 °C (sigma 3-30ks, Sigma-Aldrich, Munich, Germany).

Protein quantification of PP tissues was carried out by using Bradford reagent (Bio-Rad Laboratories GmbH, Munich, Germany) as instructed in the leaflet provided, and the EnSpire Multimode Plate Reader (Perkin Elmer, Waltham, MA, USA) was used for detection.

### 4.6. Immunoblotting and Densitometry

Protein (4.1 µg) and Laemmli buffer (Bio-Rad Laboratories GmbH, Munich, Germany) were mixed and loaded onto a 12.5% polyacrylamide gel. Electrophoresis was allowed to run for 5 min at 200 mV, followed by 60 min at 100 mV. Afterwards, the samples were transferred onto a PVDF membrane, which was blocked for 60 min in 5% milk (in Tris-buffered saline with 0.1% Tween 20) and later incubated with the antibodies for claudin-1, claudin-3, claudin-5, tricellulin, or beta-actin (Life Technologies, Carlsbad, CA, USA) overnight at 4 °C. Antibodies were used as recommended by the manufacturer (1 µg/mL). To enable visual detection, peroxidase-conjugated goat anti-mouse or anti-rabbit antibodies (Cell Signaling Technology, Danvers, MA, USA) were employed to bind the primary antibodies. Signals were then visualized by employing the chemiluminescence detection system of Clarity Western ECL Blotting Substrate (Bio-Rad Laboratories GmbH, Munich, Germany) and a luminescence imager (ChemiDoc MP, Munich, Germany).

The density of individual immunoblotting bands was analyzed by using the imager-associated software (ImageLab, BioRad, Hercules, CA, USA). The measured values were divided by the coherent beta-actin band signal. Finally, the signal for the caprate challenge was expressed in relation to the control values for VE and PP, which were set to 100%.

### 4.7. Immunohistochemistry

Tissue samples were placed into 4% formaldehyde (Roti-Histofix, Karlsruhe, Germany) for 24 h, dehydrated by using an increasing alcohol gradient, transferred to xylol, and embedded in paraffin. Paraffin blocks were cut into 3-µm-thick sections on a Leica RM 2245 microtome (Leica Microsystems Heidelberg, Germany). Before staining, the sections on the slides were warmed for 30 min at 60 °C (incubator), deparaffinized in xylol, and taken through a decreasing alcohol gradient. Depending on the protein, EDTA or citrate buffer was used for heat-induced antigen retrieval (45 min). Following the permeabilization of the samples with Triton X-100 (Carl Roth, Karlsruhe, Germany), sections were incubated with 5% goat serum in PBS and subsequently with antibodies raised against claudin-3 and -5 (1:100, Life Technologies, Carlsbad, CA, USA) for 60 min at 37 °C. After a washing step in blocking solution, samples were incubated with goat anti-mouse and -rabbit Alexa Fluor-488, and -594 (1:1000, Life Technologies, Carlsbad, CA, USA) and 4′,6-diamidino-2-phenylindole (DAPI, 1:5000) for 60 min at 37 °C. Sections were then mounted with ProTags Mount Fluor (Biocyc, Luckenwalde, Germany). Analyses and imaging of these sections was carried out by employing a Leica microscope of the DMI 6000 series (Leica Microsystems, Heidelberg, Germany).

### 4.8. Statistical Analysis

Results are always expressed as the mean ± standard error of the mean, with n being the number of animals used. For in vitro experiments, n represents the number of experiments carried out. When indicated, relative values were used for statistical evaluation.

Data from the cell culture experiments were analyzed by employing the paired Student’s t-test, whereas the results of the Ussing chamber experiments were evaluated with the unpaired Student’s t-test. Significant difference was considered at *p* < 0.05.

## 5. Conclusions

The results of this study show an increased permeability in the PP and a reorganization of the TJ structure after caprate incubation. Since caprate is a prevalent food component, these data imply a constant increasing effect on the paracellular permeability of the PP FAE. This might also accelerate the development of allergies, since more molecules are presented to the immune cells of the PP.

## Abbreviations

Cldn	Claudin
DC	Dendritic cells
FAE	Follicle-associated epithelium
GALT	Gut-associated lymphocytic tissue
IgE	Immunoglobulin E
M-cell	Microfold cell
PP	Peyer’s patch
TER	Transepithelial electrical resistance
VE	Villous epithelium
